# Clinical efficacy of combination of pertuzumab, trastuzumab, and docetaxel for treatment of patients with HER2-positive breast cancer

**DOI:** 10.1097/MD.0000000000017262

**Published:** 2019-09-20

**Authors:** Yan-cui Liu, Ying Ma, Ning An, Ping Sun, Ying Wang, Cheng Sun

**Affiliations:** a Department of Anatomy; b Department of Library, Mudanjiang Medical University; c Second Ward of Neurology Department, Affiliated Hongqi Hospital of Mudanjiang Medical University; d Institute of Neuro Tissue Engineering, Mudanjiang Medical University, Mudanjiang, China.

**Keywords:** breast cancer, docetaxel, efficacy, pertuzumab, safety, trastuzumab

## Abstract

**Background:**

This study will systematically investigate the efficacy and safety of the combination of pertuzumab, trastuzumab, and docetaxel (PTD) for treatment of patients with HER2-positive breast cancer (HER2-PBC).

**Methods:**

A comprehensive literature search for this study will consist of 2 parts: electronic database records and gray literature. The electronic database literatures are searched from PubMed, EMBASE, Cochrane Library, Web of Science, Google Scholar, Allied and Complementary Medicine Database, Chinese Biomedical Literature Database, and China National Knowledge Infrastructure. All databases will be searched from inception up to the present. In addition, gray literatures, such as dissertations, ongoing trials, and so on, will also be searched. Two authors will independently read the records, extract data collection, and evaluate the risk of bias. RevMan V.5.3 software will be applied for statistical analysis.

**Results:**

This study will summarize up-to-date evidence of PTD for patients with HER2-PBC via overall survival, complete response, cancer-specific survival, recurrence-free survival, disease-free survival, quality of life, and toxicities.

**Conclusion:**

This study will provide efficacy and safety of PTD for HER2-PBC.

## 1 Introduction

Breast cancer is 1 of the most common cancers diagnosed in females around the world.^[1–3]^ In addition, it is a heterogeneous disease with a variety of subtypes between and within tumors.^[4–6]^ The 3 major subtypes of comprehensive gene expression comprise of luminal, human epidermal growth factor receptor 2 (HER2)-positive, and basal-like cancers.^[7–9]^ Of them, the overexpression of HER2-positive breast cancer (HER2-PBC) has accounted for 15% to 20% of all patients with breast cancers.^[10]^ It has been reported that such disorder is often associated with an aggressive disease course and poor prognosis.^[11,12]^ However, its development treatment targeting on HER2-PBC has dramatically improved the outcome results and has became a landmark in the management of such patients.^[13,14]^

At present, several targeted agents are available for the treatment of patients with HER2-PBC, such as pertuzumab and trastuzumab.^[15–18]^ However, there is still restricted efficacy of such single agent. Thus, it is very necessary to apply combined single agent, including the combination of pertuzumab, trastuzumab, and docetaxel (PTD). Previous studies have reported PTD can be used to treat HER2-PBC effectively.^[19–27]^ In this study, we will systematically explore the efficacy and safety of PTD for the treatment of patients with HER2-PBC.

## 2 Methods

### 2.1 Ethics and dissemination

This study will only analyze previous completed studies, thus no ethic approval is needed. We expect to publish this study at peer-reviewed journals.

### 2.2 Eligibility criteria for study selection

#### 2.2.1 Type of studies

All randomized controlled trials (RCTs) without language limitation of PTD for HER2-PBC will be included. Non-RCTs will be excluded in this study.

#### 2.2.2 Type of participants

The patients diagnosed with HER2-PBC will be included with no limitation for the region, nation, ethnic, sex, and age.

#### 2.2.3 Type of interventions

In the experimental group, patients receiving PTD alone will be considered for inclusion.

In the control group, patients receiving any interventions, except PTD, will be included.

#### 2.2.4 Type of outcome measurements

Primary outcomes consist of overall survival and complete response. Secondary outcomes comprise of cancer-specific survival; recurrence-free survival; disease-free survival; quality of life, as assessed by any related scales; and toxicities.

### 2.3 Search methods for the identification of studies

All searched literature records consist of 2 parts: electronic databases and gray literature. The electronic databases include PubMed, EMBASE, Cochrane Library, Web of Science, Google Scholar, Allied and Complementary Medicine Database, Chinese Biomedical Literature Database, and China National Knowledge Infrastructure from inception up to the present without language limitations. Additionally, gray literatures will also be searched, including dissertations, ongoing trials, and conference proceedings. The search strategy for PubMed is showed in Table 1. Similar search strategy will also be used to other electronic databases.

**Table 1:**
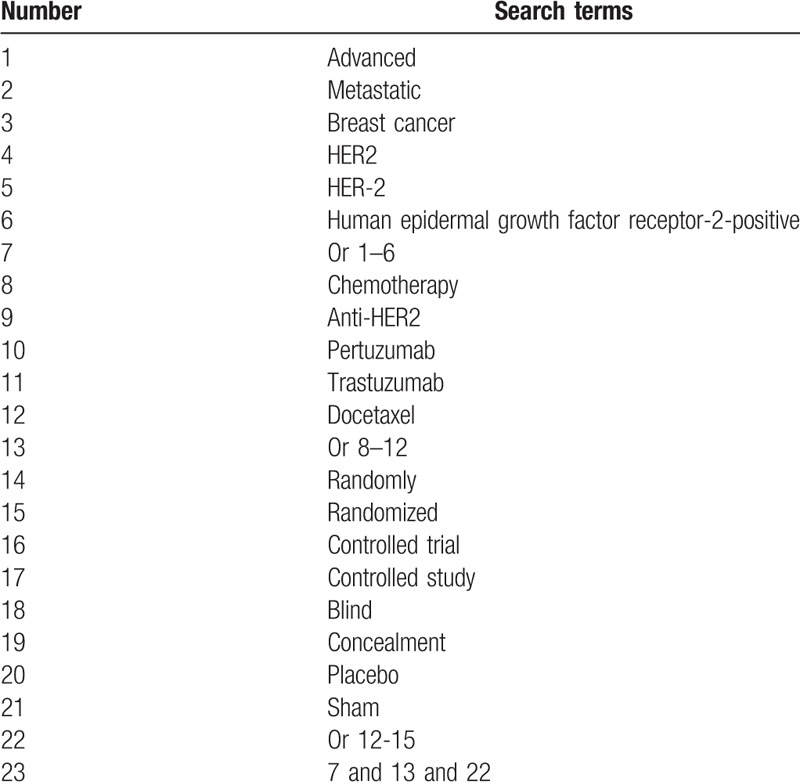
Search strategy for PubMed.

### 2.4 Data collection and management

#### 2.4.1 Study selection

Before study selection, we will build strict inclusion and exclusion criteria. Two authors independently read titles and abstracts, and also full texts, to determine whether the studies finally meet all eligibility criteria. If there are disagreements, such divergences will be decided with another author by discussion. We will present and summarize the results of study selection in the flow diagram.

#### 2.4.2 Data extraction

Two authors will independently retrieve the following data information using a predefined extraction data sheet. The information comprises of study details (title, author, year of publication, country, etc), participant details, study setting, study methods, treatment details, outcome measurements, adverse events, and any other information. Any differences between the 2 authors will be solved by a third author through discussion. If the data are unclear or not reported in the primary studies, we will contact original authors by e-mail to collect those data.

#### 2.4.3 Risk of bias assessment

Two authors will independently assess the methodological quality of each study using Cochrane risk of bias tool. It consists of random sequence generation, allocation concealment, blindness, incomplete outcome data, selective reporting, and other bias. All these items are divided into 3 levels: high, unclear, and low risk of bias. If there are disagreements, we will discuss them with another independent author to make a decision.

#### 2.4.4 Measures of treatment effect

We will calculate continuous data as the mean difference or standardized mean difference with 95% confidence interval (CI), and dichotomous data as the relative risk with 95% CI.

#### 2.4.5 Assessment of heterogeneity

Heterogeneity among included studies will be determined using *I*^2^ statistic test. When *I*^2^ ≤ 50%, heterogeneity is regarded as reasonable, and a fixed-effect model will be used. When *I*^2^ > 50%, heterogeneity is considered as substantial, and a random-effect model will be utilized.

### 2.5 Data synthesis and analysis

#### 2.5.1 Data synthesis

RevMan V.5.3 software is used to perform data synthesis. When heterogeneity is not obvious (*I*^2^ ≤ 50%), a meta-analysis will be carried out if sufficient eligible studies are included. When heterogeneity is significant (*I*^2^ > 50%), subgroup analysis will be conducted to identify any possible causes, and meta-regression analysis will also be carried out.

#### 2.5.2 Subgroup analysis

Subgroup analysis will be carried out based on the type of treatments and comparators, and different outcomes.

#### 2.5.3 Sensitivity analysis

Sensitivity analysis will be conducted to examine the robustness of pooled outcome results by removing low quality studies.

#### 2.5.4 Publication bias

Funnel plots and Egger linear regression test will be utilized to judge whether a publication bias exists if more than 10 RCTs are included in this study.

## 3 Discussion

This study will evaluate the efficacy and safety of PTD for the treatment of patients with HER2-PBC systematically. We will carry out comprehensive literature searches with no limitations of language and publication status to avoid missing more potential studies. Cochrane risk of bias tool will be utilized for the assessment of methodological quality for eligible studies. Although previous studies have assessed the efficacy and safety of PTD for HER2-PBC, its conclusion is still controversial. Therefore, this study will firstly assess its efficacy and safety of PTD for HER2-PBC systematically. We believe that the findings of this study will inform our understanding of PTD in treating HER2-PBC, and will provide helpful evidence in clinical practice.

## Author contributions

**Conceptualization:** Yan-cui Liu, Ning An, Ping Sun.

**Data curation:** Yan-cui Liu, Ying Ma, Ying Wang, Cheng Sun.

**Formal analysis:** Ying Ma, Ning An, Ping Sun, Cheng Sun.

**Funding acquisition:** Yan-cui Liu.

**Investigation:** Ying Wang, Cheng Sun.

**Methodology:** Ying Ma, Ping Sun, Cheng Sun.

**Project administration:** Yan-cui Liu.

**Resources:** Ying Ma, Ning An, Ping Sun, Ying Wang, Cheng Sun.

**Software:** Ying Ma, Ning An, Ping Sun, Ying Wang, Cheng Sun.

**Supervision:** Yan-cui Liu.

**Validation:** Yan-cui Liu, Ying Ma, Ning An, Cheng Sun.

**Visualization:** Yan-cui Liu, Ping Sun, Ying Wang.

**Writing – original draft:** Yan-cui Liu, Ying Ma, Ning An, Ping Sun, Ying Wang, Cheng Sun.

**Writing – review & editing:** Yan-cui Liu, Ning An, Ping Sun, Ying Wang, Cheng Sun.
